# Less Is More: Substrate Reduction Therapy for Lysosomal Storage Disorders

**DOI:** 10.3390/ijms17071065

**Published:** 2016-07-04

**Authors:** Maria Francisca Coutinho, Juliana Inês Santos, Sandra Alves

**Affiliations:** Department of Human Genetics, Research and Development Unit, National Health Institute Doutor Ricardo Jorge, Rua Alexandre Herculano, 321 4000-055 Porto, Portugal; juliana.santos@insa.min-saude.pt (J.I.S.); sandra.alves@insa.min-saude.pt (S.A.)

**Keywords:** substrate reduction therapy (SRT), miglustat, eligluistat tartrate, genistein, Gaucher disease (GD), Niemann-Pick type C (NPC), mucopolysaccharidosis type III (MPS III, Sanfilippo syndrome), combination therapy

## Abstract

Lysosomal storage diseases (LSDs) are a group of rare, life-threatening genetic disorders, usually caused by a dysfunction in one of the many enzymes responsible for intralysosomal digestion. Even though no cure is available for any LSD, a few treatment strategies do exist. Traditionally, efforts have been mainly targeting the functional loss of the enzyme, by injection of a recombinant formulation, in a process called enzyme replacement therapy (ERT), with no impact on neuropathology. This ineffectiveness, together with its high cost and lifelong dependence is amongst the main reasons why additional therapeutic approaches are being (and have to be) investigated: chaperone therapy; gene enhancement; gene therapy; and, alternatively, substrate reduction therapy (SRT), whose aim is to prevent storage not by correcting the original enzymatic defect but, instead, by decreasing the levels of biosynthesis of the accumulating substrate(s). Here we review the concept of substrate reduction, highlighting the major breakthroughs in the field and discussing the future of SRT, not only as a monotherapy but also, especially, as complementary approach for LSDs.

## 1. Introduction

The concept of enzyme replacement as a potential therapeutic approach to ameliorate lysosomal storage disorders (LSDs) is virtually as old as the concept of LSD itself. In fact, right after the first enzymatic deficiency underlying a LSD was described, in 1964, both concepts were established. Having reported Pompe disease as the result of a functional defect of the lysosomal enzyme α-glucosidase, Henri Hers immediately deduced that “*other deposition diseases might be explained on the basis of the absence of other lysosomal enzymes*” [1]. By doing so, he actually established the concept of LSD and its defining criteria. Quite remarkably, in that same publication, he made a theoretical prediction on the existence of congenital mucopolysaccharidoses (MPSs), lipidoses, and other storage conditions, whose existence was to be proven during the following decades [2]. At the same time, Christian de Duve, Hers’ mentor, came up with the suggestion that LSDs could be treated by replacing the defective enzymes with their normal counterparts. Briefly, de Duve explained: “*In our pathogenic speculations and in our therapeutic attempts, it may be well to keep in mind that any substance which is taken up intracellularly in an endocytic process is likely to end up within lysosomes. This obviously opens up many possibilities for interaction, including replacement therapy*” [3]. The idea itself was as simple as it could be: if an enzyme was not sufficiently active, one could try to purify it for subsequent injection into patients to check whether therapeutic benefit would be achieved. A few years after this concept was proposed, Elizabeth Neufeld’s team provided the scientific community with actual evidence of this process, reporting a cross correction phenomenon between cultured fibroblasts from patients with different LSDs [4]. The subsequent discovery that acidic hydrolases reach the lysosomes via mannose 6-phosphate receptor (MPR)-mediated pathway [5] along with the discovery that those same receptors are actually present on the plasma membrane and may mediate the cellular uptake and delivery of the intravenously administered normal enzymes to the lysosomes, provided further rationale for the treatment of this group of disorders by enzyme replacement therapy (ERT) [6].

From then on, the search for a functional version of the missing enzyme(s), either purified from human/mammalian origin or recombinantly produced has gathered most researchers’ efforts. In the early 1970s, several major obstacles seemed to hinder the development of one such approach, including: (a) the technical problems associated with massive production and purification of lysosomal enzymes; (b) the inability to target those exogenous proteins to specific tissues and cellular sites of pathology; and (c) the absence of proper animal models to allow evaluation of the pharmacokinetic and pharmacodynamic effects of enzyme administration. Brady’s group tried to overcome some of those issues by purifying a functional version of β-glucocerebrosidase (GCase, the enzyme deficient in Gaucher disease, GD) from human placentae and injecting it directly into patients [7]. Their first results were encouraging but limited in terms of clinical effects, probably due to the small dose administered [7]. Subsequently, they reported that, when the same patients were treated with intravenous (IV) infusions of larger doses of the mannose-terminated enzyme, it was possible not only to halt the progression of the disease but also to correct most, if not all, symptoms [6,8,9,10].

Unfortunately, soon the huge success achieved for type 1 GD was shown to be one of a kind. Over the following decades, several attempts were made to develop additional successful ERT approaches for other LSDs but none of them led to the spectacular effects originally seen for type 1 GD patients. Still, some enzymes have actually made their way into the market and ERT is currently available for a number of LSDs (Gaucher, Fabry, Pompe, MPS I, II, IVA and VI) or under evaluation for others. There are, however, several limitations associated with ERT. First of all, it requires lifelong IV administration, a procedure that typically exceeds €200,000 annually. At a pharmacological level, the development of immune responses limiting the efficacy of the enzyme(s) has already been reported in several patients using different preparations [11,12,13,14,15,16]. Furthermore, ERT has a limited effect in several tissues/organs. In GD, for example, even though efficient in reducing spleen and liver size and in improving anemia and thrombocytopenia, the poor distribution of infused enzyme to bone limits its effectiveness in preventing osteonecrosis, osteopenia and bone pain. In addition, pulmonary hypertension and fibrosis tend to defy ERT, as recombinant enzymes hardly reach the lungs. In Fabry disease patients, for example, ERT fails to address the renal phenotype. Finally, recombinant enzymes in general are unable to cross the Blood-brain barrier (BBB). This means that, whatever the preparation, ERT will hardly be able to cope with LSDs’ neurological symptoms. Unfortunately, brain pathology is actually one of the major burdens of LSDs, being quite prominent in the majority of the pathologies. Having this in mind, the search for therapeutic alternatives has been initiated in the recent years. Studies on gene therapy aimed at establishing an endogenous source of functional enzyme, as well as a variety of mutation-specific solutions including the use of chaperones [17,18,19], stop-codon readthrough drugs [20,21,22] and/or splicing correction oligonucleotides [23,24] are being published by different teams, some of them with promising results. Recently, the first pharmacological chaperone to be proven effective in ameliorating LSD clinical symptoms has just been approved by the European Medicines Agency (EMA) for Fabry disease management: migalastat [25,26]. Still, no matter how effective the treatment or cutting-edge the technology used in any of these cases, the underlying rationale is virtually the same: an attempt to provide or enhance the activity of the missing enzyme.

Nevertheless, an alternative approach arose 20 years now. Its theoretical basis were established in 1996, when Norman Radin came up with an academic prediction that GD patients could also be treated with a drug able to slow the synthesis of glucosylceramide (GlcCer), the lipid that accumulates in this disorder [27]. He even proposed a perfect candidate for that role: “1*-phenyl-2-decanoylamino-3-morpholino-1-propanlo (PDMP), a designer inhibitor that resembled the synthase’s substrate and product*”. By the time Radin made this prediction, it had already been shown that PDMP was “*effective in mice, rats, fish, and a wide variety of cultured cells*”. It also seemed to be “*harmless*” when used in suitable doses, even though “*long-term tests* [had] *not been made*”. Unfortunately, the lack of suitable GD animal models made it difficult to adequately test his hypothesis by the time it was published. Still, the grounds were seeded for the appearance of a second line of work on the LSDs therapeutics field, whose aim was to prevent storage not by correcting the original enzymatic defect but, instead, by decreasing the biosynthesis of the substrate that is accumulated. This approach was called substrate reduction therapy (SRT) and will be the major focus of this review.

## 2. From Concept to Clinics

### 2.1. SRT for Glycosphingolipidoses and Related Disorders

#### 2.1.1. Gaucher Disease and Other Glycosphingolipidoses

The general principle of SRT, as proposed by Radin [27], is that a small molecule drug may be used to partially inhibit the biosynthesis of the compounds, which accumulate in the absence of a specific lysosomal enzyme. By doing so, one such drug will reduce the number of molecules requiring catabolism within the lysosome, thus contributing to balance the rate of synthesis with the impaired rate of catabolism. Theoretically, this approach had a number of potential advantages when compared with ERT, including oral availability, non-immunogenicity, the use of a single compound to treat a number of diseases as well as the possibility of being able to reduce storage in the brain [28].

**Figure 1:**
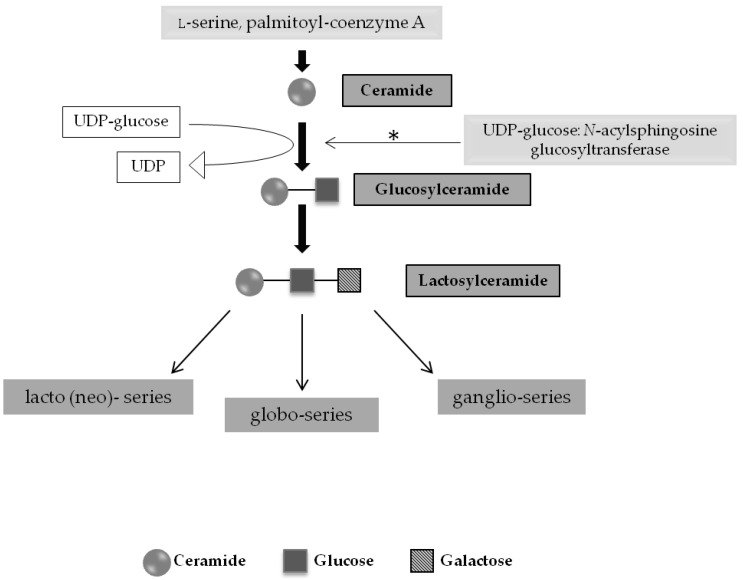
Schematic presentation of the Glycosphingolipids (GLS) biosynthesis, emphasizing the conversion of ceramide to glucosylceramide (GlcCer) via the action of UDP-glucose:*N*-acylsphingosine glucosyltransferase (ceramide glucosyltransferase, CGT), major target for substrate reduction approaches in GLS-storage diseases (*) (Adapted from [26]).

It is now known that glycosphingolipids (GSLs) are synthesized in the Golgi apparatus by the addition of monosaccharides to ceramide through the sequential action of glycosyltransferases [29,30] (Figure 1). Two main families of GSLs result: the neutral GSLs (lacto- and globo-series) and the gangliosides (ganglio-series, Figure 1) [28]. Once their cellular function is accomplished, GSLs typically re-cycle via the Golgi [31] and, as part of the normal turnover, GSLs are routed to the lysosome for degradation. This process depends on the sequential action of different glycohydrolases [32], which remove any monosaccharide from the GSsL at each step of the degradation pathway [28,33]. These enzymes, along with their activator proteins and co-factors, have been extensively characterized [34,35] and disease states associated with defective function of virtually every one of those individual proteins [36].

However, despite their exceptional value as tools to unveil the biological significance of GSLs, the compounds synthesized at the University of Michigan by Radin and colleagues in those early days aiming at inhibiting the pivotal enzyme in GSL biosynthesis, ceramide glucosyltransferase (CGT), were of little or no use for therapeutics, as limited in vivo data were obtained to support clinical trials [37]. Still, SRT did seem a promising concept and, therefore, the search for additional agents to block GSL biosynthesis has gathered other groups’ efforts. Platt and Butters, at the Glycobiology Institute of Oxford were the first team to recognize the ability of *N*-butyl-deoxynojirimycin (*N*B-DNJ) to inhibit GlcCer synthesis at low micromolar concentrations [38,39,40].

They agreed that the best way to reduce glycolipid biogenesis for therapeutic intervention would be to target an early step of the pathway and figured out that specific imino sugars could be used to block it. Imino sugars, and *N*-alkylated imino sugars in particular, are small molecule inhibitors that have an inhibitory selectivity for the enzyme that participates in the key step of the biosynthesis of all GSLs, which occurs in the Golgi apparatus [41,42,43,44]. This step refers to the conversion of ceramide to GlcCer catalysed by UDP-glucose:*N*-acylsphingosine glucosyltransferase (ceramide glucosyltransferase, CGT; EC 2.4.1.80). Imino sugar inhibitors are structural mimics of the monosaccharides where a nitrogen atom replaces the ring oxygen. This structural change provides imino sugars with a potent inhibitory activity against α-glycosidases and glucosyltransferases specific for monosaccharide hydrolysis [41,42,43]. After performing a detailed structural analysis of different imino sugars and evaluating their cell and tissue penetration, they focused attentions on *N*B-DNJ and *N*-butyldeoxygalactonojirimycin (*N*B-DGJ), which seemed the most promising molecules under evaluation.

Pivotal proof-of-principle experiments in a cell culture model of GD clearly demonstrated that *N*B-DGJ was as effective as *N*B-DNJ in preventing glycolipid storage [38,39]. Still, before clinical trials could be initiated, further studies had to be performed in adequate animal models. The first experiments were performed in a Tay-Sachs mouse model developed by Yamanaka et al. [45], as there was no satisfactory GD animal model at that time. The Tay-Sachs mouse model exhibits significant levels of storage in the brain but no clinical phenotype. Therefore, only storage could be addressed. However, the results were promising, with significantly reduced biochemical storage burden in whole brain [46]. When a second GSL storage disorder mouse model was developed by Sango and co-workers, in 1995, which was an authentic model of the human Sandhoff disease [47], *N*B-DNJ could be properly evaluated in terms of its potential impact in clinical signs and the results were excellent. In general, the pre-symptomatic period was extended, the rate of clinical decline was slowed, and life expectancy increased by 40% ([48], reviewed in [44]). If similar penetrance of the central nervous system (CNS) by *N*B-DNJ could be achievable in humans, clinical benefit seemed obvious and, thus, the next step was to perform a clinical evaluation of this compound as a SRT agent for human disease. Fortunately, *N*B-DNJ had already been studied by Searle/Monsanto as a leading candidate for HIV treatment. Clinical development culminated in clinical trials using that agent either as a monotherapy or in combination with Zidovudine, an inhibitor of reverse transcriptases. Those clinical trials were unsuccessful, as the antiviral concentrations could not be achieved [35] but the existence of a previous safety record for *N*B-DNJ eased the investigation on its possible applicability for treatment of GSLs. The first clinical study on the use of this compound to treat one of those disorders was initiated in 1998 [40,49], five years after the in vivo proof-of-principle was established for the use of *N*B-DNJ as an agent to decrease storage in a Tay-Sachs mouse model, and to ameliorate the phenotype of Sandhoff mice. In humans, the efficacy of *N*B-DNJ was first assessed in type 1 GD patients. The non-neuropathic form of the disorder was chosen over other GSL storage diseases with neurological involvement because there is a number of reasons that make clinical trials in CNS diseases more problematic: (1) smaller number of patients; (2) large clinical heterogeneity; and (3) lack of consensus on the clinically relevant endpoints and on the time needed to observe efficacy [44]. Now named miglustat (Zavesca^®^), *N*B-DNJ was evaluated for its safety and efficacy through a one-year open-label study enrolling 28 adult type 1 GD patients. At 12 months of treatment, a significant decrease of substrate formation was observed, improving key clinical features of non-neuronopathic GD [49]. An extension study from 12 to 36 months was performed and its results further supported the clinical efficacy of SRT treatment with miglustat. There was a significant decrease of spleen and liver volumes, a time-dependent reduction of chitotriosidase activity, and an improvement in hematological parameters (hemoglobin and platelet count levels). Nevertheless, some adverse side effects were also reported, including gastrointestinal tract symptoms such as osmotic diarrhea, and peripheral neuropathy [50].

In order to evaluate the efficacy and safety of miglustat, in combination with ERT, in patients with the neuronopathic form of GD, a 24-month, phase II, open-label clinical trial of miglustat in patients with GD type 3 was conducted. The study was designed in two phases: during the initial 12 months, patients were randomized 2:1 to receive either “miglustat” or “no miglustat treatment”. This randomized phase was followed by an optional 12-month extension phase in which all patients were actually given miglustat. During the 24-month period, all patients remained on ERT. Unfortunately, no improvement in the overall neurological conditions could be detected when daily miglustat treatment was attempted in GD3 patients [51,52].

Taking all this data into account, the drug was approved in 2002 by EMA and by the Food and Drug Administration (FDA) in 2003 for mild to moderate treatment of GD1. The recommended dose for this oral drug is 100 mg, three times a day [52]. The approval of miglustat as a therapeutic drug is quite remarkable, as only a few imino sugars have actually reached the clinic. Actually, until *N*B-DNJ was approved, miglitol (*N*-methoxy-DNJ, Bayer, Leverkusen, Germany) for non-insulin dependent diabetes was the sole member of this class of compounds to be in conventional use as a therapeutic drug [37].

Interestingly, when further studies were performed in order to understand the biochemical and therapeutic effects of miglustat, their results came as a complete surprise. In fact, even though having been originally conceived as a CGT inhibitor, there is now a number of papers from different laboratories showing that the beneficial effects of miglustat also have to be attributed to the inhibition of β-glucosidase 2 (GBA2), a non-lysosomal hydrolase, which degrades GlcCer. The first clues suggesting that it was not substrate reduction itself, which resulted in symptoms’ amelioration, came from studies in other glycosphingolipidoses’ mouse models, namely of Sandhoff disease and Niemann-Pick type C (NPC), where clear phenotypic effect was observed even though elevated levels of GSLs were still observed in the brain [48,53]. For those mice, treatment with a moderate dose of miglustat had significant effects on overt pathology, by increasing the lifespan, reducing CNS inflammation and ameliorating behavioral symptoms. By the time these observations were made, several hypothesis for alternative mechanisms of action were proposed for miglustat, one of them being its putative role as an inhibitor of the non-lysosomal GCase, which would ultimately result in altered levels of neuronal GSLs. Soon after these observations were made, Ridley and co-workers [54] addressed the ambiguity surrounding one of the defining characteristics of GBA2, particularly analyzing its sensitivity to inhibition by two previously reported compounds, conduritol B epoxide (CBE) and miglustat, showing that CBE inactivated GBA2 less efficiently, while miglustat exclusively inhibited the non-lysosomal GCase [54]. Recently, the notion that GBA2 inhibition could be contributing to the imino sugar’s therapeutic mechanism received further experimental support. This hypothesis was tested in two independent studies, one with a *Gba2*-deficient mouse model of type 1 GD, and the other with a *Gba2*-deficient mouse model for NPC was also inhibited. For both cases, the genetic deficiency of Gba2 had an effect similar to that of miglustat treatment, resulting in milder pathology [55,56]. Altogether, these studies raise the possibility that the inhibition of GBA2 contributes to the therapeutic potential of miglustat, in combination with its capacity to inhibit CGT. In addition, a few years ago, miglustat was also shown to act as a pharmacological chaperone towards some mutant variants of GCase [57,58].

Altogether, miglustat was a case of success and provided the proof-of-principle for the efficacy of substrate reduction strategies to achieve clinical benefit in patients suffering from LSDs. Still, it has its limitations, which are mostly related to unwanted secondary effects. In fact, even though improvements in several parameters including visceromegaly and hematological abnormalities, plasma levels of GlcCer and biomarkers of GD are observed, the extent of the response is significantly less impressive than generally observed with high-dose ERT [40]. Furthermore, the pregnancy category of miglustat is X, whereas ERTs vary from B to C: the pregnancy category of velaglucerase alfa (Shire Human Genetic Therapies, Inc., Lexington, MA, USA) and taliglucerase alfa (ELELYSO, Pfizer Inc., New York, NY, USA) is B and that of imiglucerase (Cerezyme, Genzyme Corp., Cambridge, MA, USA) is C.

Nevertheless, miglustat was not the only drug to be tested as a substrate reduction agent for GD over the last decade. The original work of Radin [27] was not set aside and, even though its’ initial results were not as promising as those observed for *N*B-DNJ, eventually persistence proved its value, with a PDMP-derived compound producing positive results. Following Radin’s rationale, the laboratory of James Shayman at the University of Michigan, focused attention on glucosylceramide analogs as inhibitors of CGT [59]. The prototype inhibitor was the original compound described by Vunnam and Radin, in 1980 [60]: PDMP (d,l-threo-1-phenly-2-decanoylamino-3-morpholino-1-propanol). It inhibited CGT at micromolar concentrations but had little specificity toward the enzyme [60,61]. This original compound had three primary functional groups: a cyclic amine, an aromatic group, and a fatty acid in amide linkage. Over the years, the Michigan team has subsequently undertaken a systematic substitution of those groups to identify potential glycolipid synthesis inhibitors and they ended up developing more specific and potent drugs. The most promising analogs were p-OH-P4 (d-threo-1-4′-hydroxyphenyl-2-palmitoylamino-3-pyrrolidino-propanol) and EtDO-P4 (d-threo-1-ethylendioxyphenyl-2-palmitoylamino-3-pyrrolidino-propanol) [61,62,63,64]. In vivo proof-of-concept studies for the use of those compounds were initially performed in Fabry disease models, in which globotriaosylceramide accumulates in the vasculature and kidney due to a loss of β-galactosidase A activity. Those models were chosen because, at that time, the only suitable mouse model for GD was associated with early neonatal death. EtDO-P4 did also significantly reduce the GlcCer and globotriaosylceramide content of transformed lymphoblasts from Fabry disease patients [63,64]. Further evaluation of EtDO-P4 predicted it to have a very high degree of hydrophobicity. Thus, despite its excellent activity, soon Shayman and his team [65] were evaluating the effect of fatty acyl chain substitutions of EtDO-P4. The most suitable pharmacokinetic profile was detected for the C8-substituted homolog, eliglustat tartrate, with limited loss of inhibitory activity [65]. Pre-clinical pharmacological studies in normal mice, rats and dogs with IV and oral administration, as well as those performed in a knock-in GD mouse model (gbaD409V/null, also known as 4L;C*), demonstrated that the agent had a high therapeutic index, excellent bioavailability and limited toxicity [65,66,67]. In order to further assess the potential therapeutic effect of this compound, a collaborative series of enabling studies were pursued between the Genzyme and Michigan groups [61] and, over the following five years, eliglustat tartrate (Genz-112638) and its free base (Genz-99067) were the subject of several clinical trials. Phase I studies assessed safety, tolerability and pharmacokinetics in escalating single and multiple doses [68] and, soon after their results were released and clinical safety of the compound demonstrated, an open-label, single arm phase II clinical trial was initiated [61,65]. The results of both studies were published in detail and, overall, they showed that eliglustat tartrate was safe and effective: in healthy volunteers, plasma GCase concentrations were decreased after oral dosing with the drug and, in open-label phase II clinical trials in patients with GD1, impressive hematological responses were detected together with significant decreases in spleen and liver volumes [69,70,71,72,73]. In general, the primary outcomes of organ size reduction and improvements in hematological parameters were either comparable or exceeded those observed with imiglucerase, while clearly exceeding those reported for miglustat. Additionally, clinical data collected after eliglustat dosing regarding skeletal pathology were quite encouraging [74]. This is particularly important since bone complications have been largely refractory to ERT. In addition, both the data on efficacy and safety of eliglustat tartrate did seem superior to those reported for miglustat, the latter agent showing less significant responses to clinical outcomes and a significantly less favorable profile of untoward effects [61,73]. Finally, the results of controlled clinical trials in adult patients with non-neuronopathic Gaucher disease naïve to specific treatment compared with placebo (“ENGAGE”) and in another non-inferiority trial (“ENCORE”) enrolling patients whose disease had already been controlled by ERT (mostly imiglucerase) and were stable for at least three years, changing to eliglustat, also confirmed that this drug has a therapeutic effect, which rivals that of ERT. These two pivotal phase III clinical trials are now in their extension phases [73,75].

Therefore, eliglustat tartrate (Genzyme Corp.) was approved by FDA in 2014 for the long-term treatment of adult patients with the type 1 form of GD. The marketing authorization throughout the European Union was given by EMA in 2015. The recommended dose for this oral drug is 100 mg, three times a day [52].

Since the initial proof-of-concept studies were performed in Fabry disease models, patients suffering from this disorder should also be considered for eliglustat tartrate therapy. Nevertheless, given its poor CNS-penetration, other LSDs including Tay-Sachs disease, GM1 Gangliosidosis and the neuronopathic sub-types of GD (types 2 and 3), will still require the development of eliglustat homologs, which are able to cross the BBB [65,76]. This is an urgent need since neither ERT preparations nor the SRT drugs available so far, are CNS-accessible. Consequently, a number of different compounds and alternative approaches are still being investigated to address the CNS pathology. Alternative approaches include gene therapy and a variety of efforts to reconstitute active GCase particularly in the CNS either by transplantation of bone marrow or hematopoietic stem cells or by direct delivery of the enzyme into the brain. Small-molecule drugs are also being evaluated, either as chaperones or as novel SRT agents. Genzyme Corporation, in particular, has been working in newer PDMP-based compounds for clinical development and, over the last years, exciting news have come out of their laboratories. The first innovative molecule to be reported, after the successful approval of eliglustat tartrate as an SRT drug for GD type 1, was GZ161 ((*S*)-Quinuclidin-3-yl(2-(2-(4-fluorophenyl)thiazol-4-yl)propan-2-yl)carbamate). Pre-clinical tests in the K14 mouse, a murine model of acute neuronopathic GD, which presents with a complete loss of GCase activity, were quite successful. Using this severe disease model, Cabrera-Salazar and co-workers observed a decrease in the accumulation of glucosylsphingosine and GlcCer associated with ameliorated gliosis (including signs of neuroinflammation and infiltration of macrophages and microglia) and prolonged survival, after systemic administration of the inhibitor, clearly demonstrating the ability of the compound to cross the BBB [77].

More recently, thought, two other compounds were assessed for their efficacy to treat brain disease: GENZ-682452 and GZ/SAR402671. Both are in clinical development for Fabry disease and GD type 3, having GZ/SAR402671 received FDA fast-track designation for Fabry disease early in 2015. In both cases, two independent GD mouse models were used for in vivo assays: the conduritol β epoxide (CBE)-induced mouse model of neuronopathic GD [78] and the genetic 4L;C* model [79]. The results for GENZ-682452 have just been published and show that both models displayed nice results concerning the extent of glycolipids accumulation. In the CBE-induced mouse model, GENZ-682452 reduced the accumulation in liver and brain, while ameliorating the extent of gliosis and severity of ataxia; in the 4L;C* mouse model, GENZ-682452 further reduced the levels of substrate in the brain, while reducing the extent of gliosis and paresis. Apart from these promising results on biochemical and histological parameters, two other observations are also worth mentioning: a partial behavioral aberration correction and an increase of lifespan were also seen after treatment with GENZ-682452 of CBE-induced and 4L;C* mouse models, respectively [80]. The results for GZ/SAR402671, even though still unpublished, have been presented early this year, at the Brains4Brain society meeting [81]. In the 4L;C* mouse, oral administration of that CNS-accessible CGT inhibitor delayed both CNS histopathologic findings and substrate accumulation with a concomitant ~40% increase in lifespan. Similar results were obtained for the CBE-induced mouse model, where GZ/SAR402671 administration resulted in attenuation of all the neuropathologic manifestations including astrogliosis, microgliosis, substrate accumulation and ataxia. Once again, when taken together, these results strongly support SRT as a feasible and advantageous treatment of neuronopathic GD.

Altogether, these findings indicate that SRT using improved or modified CGT inhibitors that have brain access may represent an effective approach to treat the neurological symptoms in patients with GD type 3, further strengthening the belief that potent systemically administered CGT inhibitors that transverse the BBB are likely to be brought to patients in a near future. Still, it is important to notice that measuring clinical efficacy in type 3 GD patients presents many challenges, including the large spectrum of symptoms together with their variable degree of severity and the variable rate of disease progression, the irreversible nature of the neurodegenerative changes, and the paucity of CNS-related biomarkers that could serve as a surrogate endpoint [80]. Finally, whenever these difficulties are surpassed and one such compound becomes available as a successful therapeutic strategy for a neuronopathic glycosphingolipidosis, its use may be extended to include other related pathologies where GSLs also accumulate such as late-onset Tay-Sachs and Sandhoff diseases. This revolutionary idea that a single orally available compound could ameliorate disease by decreasing the overall GSL levels, prompted additional studies in different pathologies where those same compounds are accumulated. Since the biochemical pathway which results in the synthesis of gangliosides is preceded by the synthesis of GlcCer, inhibition of the production of the latter compound could potentially be employed in the treatment of virtually every disease caused by defects in either ganglioside or globoside degradation. Defects in ganglioside degradation, or gangliosidoses, are a subgroup of LSDs to which belong GM1 gangliosidosis, Tay-Sachs and Sandhoff diseases; defects in globoside degradation include other glycosphingolipidoses, such as Fabry disease [10]. It seemed, though, quite tempting to test the use of a single drug to treat all those GSL storage diseases. The potential advantages were multiple and obvious. Therefore, over the years, several studies were performed to assess the efficacy of a SRT drug to ameliorate the phenotype of the majority of these disorders. As *N*B-DNJ was the first compound under clinical development and the one that first reached the market, most of the studies in animal models were performed using this drug as the putative substrate reduction agent. Efficacy was demonstrated in mouse models of GM1 gangliosidosis [82,83], Tay-Sachs [46], Sandhoff [84,85] and Fabry diseases (Heare and Platt, unpublished results) [28]. Consequently, *N*B-DNJ was predicted to be of therapeutic benefit, at least for the juvenile and adult onset variants of these disorders. The infantile onset variants will most certainly require additional enzyme augmenting modality for the pathology to be significantly improved [28].

Another important factor that has to be taken into account is the major site of pathology. Different disorders have different storage locations and, therefore, pathology arises in different tissues and/or organs. In general, whatever the therapeutic option under evaluation, visceral storage sites are more accessible than the neurological ones. The major site of accumulation in gangliosidoses, Sandhoff and Tay-Sachs diseases, for example, is neural tissue, with many patients presenting with acute neurodegeneration that often leads to premature death [37]. In Fabry disease, on the other hand, death occurs due to cardiovascular disease and/or liver failure, as a consequence of a massive cardiac and renal storage of GSLs [37]. Still, as in every other LSD, glycosphingolipidoses have considerable symptomatic heterogeneity, with a broad phenotypic spectrum having been described for each one. The degree of pathology depends on the mutational impairment of catalytic activity, with low levels of enzyme activity usually predicting that symptoms’ onset occurs more rapidly leading to infantile or juvenile disease. Nevertheless, the fact that severe neurological phenotypes are relatively common in all these disorders should always be taken into account and the disappointing results on the use of *N*B-DNJ to the neuropathic should not be ignored. Furthermore, *N*B-DNJ has multiple activities against enzymes involved in glycoconjugate biosynthesis and catabolism, a feature that considerably limits dose escalation. At high doses, it causes several side-effects, as already referred for GD. Thus, work still needs to be done before moving into clinics. Once again, different compounds may be evaluated. Fran Platt’s lab, at Oxford, focused attentions on the identification of *N*B-DNJ-related compounds with greater selectivity and ended up selecting *N*B-DGJ as the most promising one [28]. In the meantime, the outstanding results obtained with eliglustat for the non-neuronopathic form of GD, also place it as a potential candidate for SRT in GSL storage diseases. Nevertheless, none of these compounds has reached clinical trials for other disorders than GD1, even though individual case reports have proven it may be beneficial in other human glycosphingolipidoses patients [86].

#### 2.1.2. Niemann-Pick Type C

Evident successful therapeutic effect, however, was actually achieved in a disorder that does not belong to the complex glycosphingolipidosis family: Niemann-Pick type C disease (NPC), another LSD affecting the brain, in which disturbed cholesterol trafficking to lysosomes is associated with a secondary accumulation of gangliosides and other GSLs (and possibly sphingosine) in neurons [87]. Even though being an apparently secondary event, in 2001 Zervas and co-workers [88], hypothesized that this buildup of GSLs could be centrally involved in the pathogenesis of NPC disease. To evaluate that possibility, they treated murine and feline NPC models with miglustat. Remarkably, treated animals showed delayed onset of neurological dysfunction, increased the average life span in the mouse model, and reduced ganglioside accumulation and accompanying neuropathological changes [88]. Apart from opening up novel avenues for laboratory investigation of NPC pathophysiology, these results have also prompted additional studies on the potential use of this drug as a therapeutic option for this disorder. Soon a prospective clinical trial was carried out and its results appeared to show neurological stabilization or even benefit in a few NPC individuals, with improvement in supranuclear gaze palsy and dysphagia [73,89]. Since then several case series on long-term miglustat therapy for NPC patients have presented clinical findings supporting the idea that miglustat is an appropriate agent to stabilize the disease [73,90,91,92,93]. Thus far, it has been approved as a treatment for adult and paediatric NPC patients with progressive neurological manifestations in 43 countries, including the European Union since 2009 and Japan since 2012. However, approval of this innovative agent has not, at the time of writing, been granted in the USA by FDA, who declined to approve it in 2010 and called for more data. Nevertheless, it should be noticed that, at least while no definitive treatment is developed for this relentless neurodegenerative disorder, miglustat or any equivalent inhibitor of GSL synthesis may be the only drugs, which deliver relief from the accelerated clinical decline seen in some patients with this cruel dementing illness [73]. Recently, Santos-Lozano and co-workers [94] have performed a systematic review on later findings of clinical trials, further supporting that miglustat can slow the progression of neurological symptoms in all evaluated patients. Nevertheless, the authors have drawn attention to the fact that there is no uniformity among published trials in the presentation of results: the time course of the disease (i.e., percent of improvement stagnation or deterioration) was assessed through different neurological parameters (horizontal saccadic eye movements, HSEM; cognition; ambulation; swallowing). Also worth mentioning, the gathered data clearly showed that the therapeutic benefit is greater in those with a late diagnosis (i.e., late childhood onset or juvenile/adult onset) compared with early childhood onset [94].

### 2.2. Mucopolysaccharidoses—Special Focus on MPS Type III (Sanfilippo Syndrome)

Glycosphingolipidoses were not the only LSDs to be tested for SRT. In fact, lysosomal diseases involving the storage of GlcCer-derived GSLs are far from being the only pathologies of the group that present with severe neurological symptoms. Other sub-groups exist, which are particularly well known for their neurological symptoms. Mucopolysaccharidoses (MPSs) are among those. MPSs comprise a series of different disorders, characterized by progressive accumulation of glycosaminoglycans (GAGs), being caused by their impaired degradation. The CNS dysfunction-related symptoms occur in most MPS I (Hurler subtype), MPS II and MPS VII patients, as well as in all MPS III patients, where they are especially severe [95]. MPS type III, also known as Sanfilippo syndrome comprises a group of four conditions (MPS III A, B, C and D), which result from individual genetic deficiencies in different enzymes involved in the degradation of herapan sulfate (HS). All four subtypes present similar clinical symptoms: severe learning difficulties and behavioral disturbances associated with mild somatic disease. Onset of clinical features usually occurs between two and six years of age, severe neurologic degeneration occurs in most patients between six and 10 years, and death occurs typically during the second or third decade of life [96,97].

**Figure 2:**
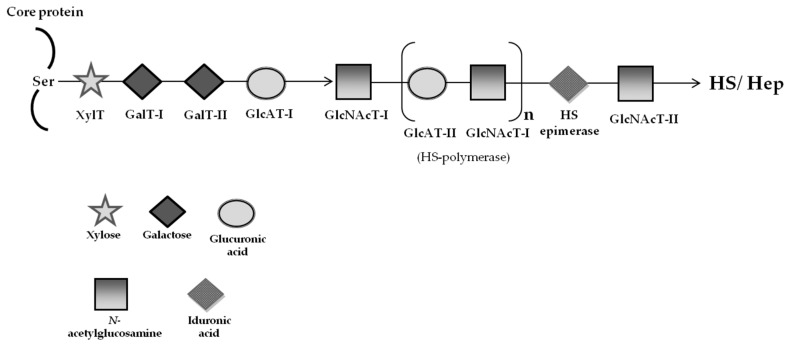
Schematic presentation of the biosynthetic assembly of heparan sulfate (HS) and heparin (Hep) from GAG backbones through the action of several glycosyltransferases (adapted from [106]). Each glycosyltransferase requires the respective UDP-sugar as a donor substrate. Following the synthesis of specific core proteins, the synthesis of the so-called GAG-protein linkage region, GlcUA β1—3Gal β1—3Gal β1—4Xylβ1-O-, common to chondroitin sulfate/dermatan sulfate (CS/DS) and HS/Hep chains, is initiated by XylT, which transfers a Xyl residue from UDP-Xyl to the specific Ser residue in the endoplasmic reticulum, and is completed by the consecutive addition of each sugar by GalT-I, GalT-II, and GlcAT-I, which are common to the biosynthesis of both CS and HS, in the Golgi apparatus. The addition of 1–4-linked GlcNAc to the linkage region by GlcNAcT-I initiates the assembly of the HS repeating disaccharide region, (-4GlcNAcβ1—4GlcUAβ1-)_n_. Then, the chain polymerization of the HS chain is catalyzed by HS-GlcAT-II and GlcNAcT-II activities of HS polymerase, which is a heterocomplex of EXT1 and EXT2. After the formation of the heparan backbone, GAG chains are matured by sulfation at various positions and epimerization at GlcUA residues. Each enzyme (glycosyltransferase and/or epimerase) is described by its respective sugar symbol: β-xylosyltransferase (XylT); β-1,4-galactosyltransferase-I (GalT-I); β-1,3-galactosyltransferase-II (GalT-II); β-1,3-glucuronosyltransferases (GlcAT-I and GlcAT-II); and 1,4-*N*-acetylglucosaminyltransferase (GlcNAcT-I). Sulfotransferases involved in the chain modifications are not included.

Being diseases that primarily affect the brain and nervous system and since neither bone marrow transplantation (BMT), nor ERT can be effective in treating their neurological symptoms, MPSs type III were considered a perfect target for the development of SRT approaches. However, in the process leading to the synthesis of GAGs the building blocks are carbohydrate or their derivatives (e.g., galactose, xylose, *N*-acetylglucosamine and others), compounds that are also involved in a huge variety of metabolic pathways (Figure 2). Therefore, an analog of such compounds working as a functional competitor would most probably interfere with many metabolic pathways, by blocking other biochemical reactions. The side effects of one such approach seemed obvious (and potentially serious). Having this in mind, Piotrowska and colleagues [98] designed an alternative SRT approach based on the regulation of expression of genes encoding specific enzymes involved in the biosynthesis of GAGs. Considering that maximum synthesis of HS and dermatan sulfate (DS) requires follicle-stimulating enzyme hormone and epidermal growth factor (EGF), these authors hypothesized that GAGs’ synthesis would be inhibited by any drug, which actively promoted a decrease of its activity. EGF influences gene expression by binding to its transmembrane receptor, thus triggering a specific kinase cascade, which ultimately results in a fine regulation of several transcription factors. Knowing that the tyrosine-specific protein kinase activity of the EGF receptor is inhibited by genistein [4′,5,7-trihydroxyisoflavone or 5,7-dihydroxy-3-(4-hydroxyphenyl)-4H-1-benzopyran-4-one], a chemical from the group of isoflavones, they assumed that this drug would actively reduce GAGs’ synthesis [98]. Initial studies in fibroblasts of patients with various forms of MPSs, namely types I, II, IIIA and IIIB were actually quite promising, since genistein was shown to inhibit the synthesis of GAGs. Most importantly, they have also observed a clear reduction in GAG storage in affected cells, which could either be due to degradation of accumulated GAGs by residual activity of the deficient enzyme, or dilution of GAGs in fibroblasts as cells divide [98]. Moreover, it was verified that genistein was able to cross the BBB and to decrease urinary and tissue GAG levels in vivo when MPS II mice were treated with that isoflavone. After 10 weeks of treatment, urinary GAG levels were decreased and, in some animals, even GAG deposits in brain were reduced by genistein treatment [99].

Similar results were obtained by an independent team working with another inhibitor, rhodamine B ((9-(2-carboxyphenyl)-6-diethylamino-3-xanthenylidene)-diethylammonium chloride). Remarkably, in MPS IIIA mice treated with rhodamine B, GAG storage decreased not only in somatic tissues, but also in brain, with improved behavior of the animals [100,101]. Later, a trans-generational study was performed to evaluate the continuous exposure of rhodamine B treatment in MPS IIIA mice over 4 generations, including treatment during pregnancy. No alterations in litter size, liver histology or liver function were observed. Overall, there were no long-term issues with the administration of rhodamine B at the low dose tested and no adverse effects were noted during pregnancy in mice [102].

Altogether, these encouraging results lead to the development of an open-label pilot clinical study with 10 children suffering from Sanfilippo syndrome types A and B in which a genistein-rich isoflavone extract (SE-2000, Biofarm, Wałbrzyska, Poland) orally administered for 12 months at the dose corresponding to 5 mg genistein per 1 kg of body weight daily. After one year of treatment, statistically significant improvement in all tested parameters was demonstrated: urinary GAG levels were reduced, hair morphology was improved and scores in the psychological test were higher. Also noteworthy, no adverse effects were noted [10,103]. A subsequent two-year follow-up of eight of those patients reinforced the idea that a genistein-rich soy isoflavone extract may be effective in either inhibition (in some patients) or slowing down (in other patients) of behavioral and cognitive problems over a longer period. An increased dose of genistein was also suggested to improve the efficacy of the treatment noted [104]. Improvement in the range of joint motion in seven patients with MPS II during experimental gene expression-targeted isoflavone therapy (GET-IT) was also reported [105].

Unlike genistein, rhodamine B never got to be tested at a clinical level, as there are serious problems which prelude the use of this compound as a drug for humans. First, rhodamine B appears to be a non-specific inhibitor of GAG synthesis and secondly, and perhaps most importantly, hazardous effects of acute exposure to rhodamine B in humans had already been reported and include mucous membrane and skin irritation [107], even though the effect of long-term exposure is unknown [95]. Also noteworthy, the mechanism by which rhodamine B reduces GAG synthesis remains elusive. In the meantime, though, studies on the mechanism of genistein-mediated inhibition of GAG synthesis have confirmed the theoretical prediction that the main regulatory pathway affected in this biological system is the signal transduction pathway initiated by the protein phosphorylation reaction stimulated by EGF interaction with its receptor [95,108]. Recently, it has also been demonstrated that genistein enhances expression of genes coding for GAG hydrolases and stimulates lysosomal biogenesis and function through modulation of transcription factor EB (TFEB) expression and activity [58,109].

Considering the encouraging experimental results of genistein treatment, some authors are also proposing it as a potential therapeutic option for other LSD where secondary accumulation of GAGs occurs (e.g., mucolipidoses, multiple sulfatase deficiency). It is, though, possible that additional studies using genistein and/or other flavonoids appear in the near future [95].

## 3. A Step Forward: Second Generation Compounds and Genetic SRT

Two decades after their rationale was first established, some SRT drugs have already been approved (miglustat and eliglustat tartrate for GD) or are undergoing clinical trial (genistein for MPS) [10,87,110]. The recognition of the pivotal role that storage may play in disease pathology together with the numerous observations demonstrating that reducing substrate levels may be of therapeutic benefit have also prompted the search for highly specific substrate clearance drugs such as cyclodextrin, which is being evaluated for NPC treatment, or cysteamine, which disperses cysteine deposits in cystinosis by forming soluble mixed-thiols [87]. Nevertheless, chemical drugs always have their side effects, as evidenced by the osmotic diarrhea and weight loss caused by miglustat. In order to overcome this issue, second generation SRT compounds are being evaluated for their potential to reduce GSLs’ storage. The group who originally reported miglustat (*N*B-DNJ), is now evaluating a second compound that had also been identified in the original screen of imino sugars with inhibitory properties against CGT: *N*B-DGJ [39]. This compound is equivalent to miglustat in terms of potency, but lacks many of the additional enzyme inhibitory properties associated with *N*B-DNJ [44,111,112]. Therefore, it does not inhibit gut disaccharides, the property of miglustat that underlies osmotic diarrhea. Additionally, *N*B-DGJ does not cause weight loss in mice, which may be an advantage particularly in the treatment of pediatric patients. Those advantages have made this compound a good candidate for high-dose human therapy, and *N*B-DGJ was recently licensed by Actelion, being under clinical development for Fabry disease. Phase I clinical trial started in June 2015 and is now completed, with no published results up to date.

Also under consideration is a totally molecular, drug-free approach to selectively downregulate genes involved in the biosynthesis of accumulating substrates, based on a promising gene suppression technology: RNA interference (RNAi). This approach has recently been referred to as genetic SRT (gSRT; [58]). Being based on a naturally occurring post-transcriptional gene silencing process, the RNAi mechanism has several advantages when compared to other gene suppression methodologies (antisense oligonucleotides or ribozymes for example). RNAi therapeutic applications are emerging, particularly in the fields of oncology, viral infections, diabetes, cardiovascular, bone-related and ocular diseases [113]. Thus far, however, reports on its use to attempt substrate reduction are scarce, even though the possibilities are multiple and obvious. It has already been used in the LSD field to inhibit the genes implicated in GSL metabolism [114]. Later, it was tested for the ability to inhibit expression of the GlcCer synthase gene as a SRT for GD [115]. Other interesting results were obtained when small interfering RNAs (siRNAs) were used to reduce GAG synthesis in MPS IIIA mice [116] and, more recently, in MPS IIIC patient cells [117].

Despite the successes achieved in gene silencing, as any other technology, RNAi does have some limitations. For example, it can only promote an incomplete inhibition of the targeted gene. In the LSD field, however, rather than a con, this is actually a pro, since the ultimate goal is to reduce the overall levels of accumulating substrates but never to completely abolish them, as those compounds have a role to play in normal tissue and organ function. Furthermore, RNAi approaches offer the possibility to establish a rationale, easy-to-follow and relatively economical pipeline to design and test SRT for multiple LSDs. Amongst other advantages, one such approach would virtually allow for the creation of multifunctional complexed si/shRNA mixtures, as different diseases share the same accumulating substrates. Therefore, instead of a “one-compound-to-one-disease” approach, SRT by RNAi may pave the way for a “one-compound-to-treat-several-diseases” era, reducing therapy costs and increasing the number of patients with available therapeutic options. Still, translation into clinics requires two major developments: proper vectors for in vivo deliverance and suitable animal models to test the approach before trials. Currently, there are a number of animal models for different LSD, most of them nicely mimicking patients’ symptoms [118]. Nevertheless, most studies have been undertaken in small rather than large animal models, even though an increasing number of those models are being identified lately. Delivery, however, remains a challenge, with several teams currently evaluating different approaches to achieve increased bioavailability and optimal tissue targeting of these therapeutic approaches. CNS-targeting remains the ultimate challenge, especially when most formulations are adequate for systemic administration but there is an actual need to cross the BBB.

## 4. Conclusions

The complex pathophysiology of LSDs and their phenotypic variability challenge the potential for single agents to effectively treat all the aspects of LSD. Combination therapies allow for a more personalized care program for patients [76,119]. Some years ago, a report by Capablo and co-workers [120] described the results of a combination of ERT (imiglucerase, Cerezyme^®^) with SRT (miglustat, Zavesca^®^) in a patient with the neuronopathic form of GD (type 3), who, despite good visceral and analytical response to ERT, developed marked myoclonic epilepsy and dystonia. After two years of such a combined therapy, generalized tonic-clonic seizures decreased, while speech and general neurologic status improved [10,120]. Right after this promising report, Cox-Brinkman and co-workers [121] have also published the clinical findings of three siblings with GD type 3, who received different therapeutic regimens during the course of their disease, including ERT monotherapy (imiglucerase; Cerezyme^®^), switch from ERT towards combined ERT and SRT therapy, as well as direct and early initiation of this combined therapy (in the youngest sibling). In general, their results added to the idea that a combination therapy may be beneficial for GD type 3 patients, with the youngest sibling presenting an almost normal neurological development, while the others developed convergent strabismus, cognitive decline and abnormal electroencephalography (EEG) and brainstem auditory evoked response (BAER) [121]. To the best of our knowledge, these are the only clinical reports on the effects of combined ERT/SRT, but their results do seem quite promising for neuronopatic forms of GD, further encouraging additional exploration. In addition, in GD mouse models, combined ERT/eliglustat treatment suggested a synergistic effect of both therapies [122].

The relevance of a synergistic effect of combined ERT/SRT is particularly evident for disorders in which most patients are have no enzymatic activity at all. For Fabry disease, for example, most male patients are null for α-galactosidase A activity. In those cases, SRT is unlikely to be effective treatment as a monotherapy. The SRT approach proposed for Fabry disease, uses inhibitors of CGT (which catalyzes the first step in the synthesis of GSLs, GL-1), limiting the production of subsequent molecules including GL-3 [123]. In Fabry disease mouse models, when combined in vivo with ERT, an increased therapeutic benefit was obtained, both additive and complementary. This can bring a treatment option allowing for a reduced frequency of ERT while on SRT maintenance therapy, potentially improving quality of life through a reduced dependency on enzyme infusions [58].

It is important to notice that ERT/SRT is not the only possible combination approach in the LSD field. In fact, most LSDs have no ERT available. Therefore, different authors have been trying to associate the most efficient SRT compounds available so far with other treatments, which have either been approved or are under evaluation. For Sandhoff disease, for example, the efficacy of SRT combined with BMT was demonstrated in vivo in studies with knockout mouse models [101,124]. Additionally, a combination of miglustat with a ketogenic diet was recently attempted in a six-year-old male patient with Sandhoff disease, with improved outcomes [125]. One such approach was designed since it had already been shown that: (a) SRT by miglustat might be useful to stabilize the neurological effects of the juvenile and adult forms of the disease [126]; and (b) a ketogenic diet resulted in improved motor behavior and longevity in diseased mouse models [127]. Another therapeutic strategy, which has been tested recently, is to combine SRT (miglustat) with curcumin and ibuprofen, two nonsteroidal anti-inflammatory drugs (NSAIDs). This approach has been tested on Niemann-Pick type C1 mice and was based on the rationale that miglustat will target sphingolipid synthesis and storage, while curcumin may compensate lysosomal calcium defect and ibuprofen reduce CNS inflammation. Such triple combination therapy had a greater neuroprotective benefit than mono- or dual-therapy [58,128] even though it has been suggested that the specific kind of SRT based on genistein and called gene expression-targeted isoflavone therapy (GET-IT), could be used together with ERT for MPS treatment, to date only preliminary experiments with that combination treatment on MPS I cell cultures have been reported [129]. Nevertheless, their results have clearly suggested that such approach may be effective [58]. However, setbacks were also observed. For instance, when SRT with miglustat was tested in combination with neuronal stem cells transplantation, no synergistic effect was observed [89].

Finally, it is important to notice that there are also several studies demonstrating that SRT may be an effective treatment by itself and should be considered for other neurodegenerative diseases caused by storage of certain compounds [130]. This is particularly important for LSDs without ERT available such as the neurological forms of MPSs, where the therapy based on the impairment of synthesis of compounds that cannot be degraded efficiently in cells was the first to show some efficacy in clinical studies.

## Appendix A

### A1. Further Reading

#### A1.1. On the Individual SRT Drugs

For a critical overview on the biochemical and molecular impact of miglustat (*N*B-DNJ) and its potential as a therapeutic drug, see [131].

For a personal and nicely detailed overview on the development of eliglustat tartrate as a therapeutic drug for Gaucher disease type 1, see [65].

For an extensive and updated overview on glycosphingolipidoses and their available and potential therapeutic approaches followed by a revision on disease pathophysiology see [76].

For an overview on the use of substrate reduction therapies for Mucopolysaccharidoses and the putative biological mechanisms underlying its efficiency, see [130,132], respectively.

#### A1.2. On LSD and Their Therapeutic Possibilities

For a comprehensive overview on innovative treatments for lysosomal storage disorders, either addressing primary pathology causes, or rebalancing the effects of disordered cell function, see [73].

For an exhaustive and up-to-date literature review on the use of combination therapy as a treatment for lysosomal storage disorders, see [58].

Finally, for those who seek an up-to-date accessible volume addressing both the scientific and the clinical aspects of lysosomal storage diseases, either as a group or as individual disorders, see Lysosomal Storage Disorders, a practical guide, edited by Athul Mehta and Bryan Winchester in 2012 [133].
