# Coutinho et al. Less Is More: Substrate Reduction Therapy for Lysosomal Storage Disorders. *Int. J. Mol. Sci.* 2016, *17*, 1065

**DOI:** 10.3390/ijms18010178

**Published:** 2017-01-17

**Authors:** Maria Francisca Coutinho, Juliana Inês Santos, Sandra Alves

**Affiliations:** Department of Human Genetics, Research and Development Unit, National Health Institute Doutor Ricardo Jorge, Rua Alexandre Herculano, 321 4000-055 Porto, Portugal; juliana.santos@insa.min-saude.pt (J.I.S.); sandra.alves@insa.min-saude.pt (S.A.)

The authors wish to make a change to their published paper [1] and add novel references.

On page 7, second paragraph, the authors stated that “the recommended dose for this oral drug [eliglustat tartrate] is 100 mg, three times a day”. Nevertheless, this dose regimen refers to miglustat, the other SRT drug currently approved for type 1 Gaucher disease. The management recommendations are different for eliglustat. Eliglustat is a CYP2D6 and CYP3A substrate. Thus, its co-administration with drugs that inhibit CYP2D6 and CYP3A may significantly increase the exposure to eliglustat and result in prolongation of the PR, QTc, and/or QRS cardiac interval, which could result in cardiac arrhythmias [2]. In brief, the recommended dose of 84 mg eliglustat twice daily for extensive and intermediate metabolisers is reduced to 84 mg once daily in poor metabolisers. Still, it is important to notice that, because of its genetically determined metabolism in the liver, eliglustat requires individual adaptation of the dose and careful supervision of concomitant medications [3].

On page 10, the authors refer to an in vivo study using MPS II mice, where a 10-week genistein treatment resulted in decreased GAG deposits in brain. An important reference, however, has been inadvertently omitted in the appended review. In 2009, Malinowska and co-workers [4] demonstrated decreased lysosomal storage in peripheral tissues of a mouse model of Sanfilippo syndrome B (Mucopolysaccharidosis IIIB) treated with genistein. Soon after this first study, the same team provided proof of concept for that isoflavone working as an SRT in the brain of the same mouse model by demonstrating improved synaptic vesicle protein expression and secondary storage in the cerebral cortex [5]. Considering those results, a phase III double blinded, randomised, placebo controlled clinical trial of high dose oral genistein aglycone in patients with Sanfilippo syndrome was designed, and is currently ongoing in Manchester [6]. Even though its outcomes have not been published to date, it has been widely reported in the field and at MPS meetings.

The authors apologize for any inconvenience. The manuscript will be updated, and the original will remain online at the article webpage.
